# Minimally Invasive Aortic Valve Replacement in Elderly Patients: Insights from a Large Cohort

**DOI:** 10.3390/jcm15010354

**Published:** 2026-01-02

**Authors:** Lukman Amanov, Arian Arjomandi Rad, Sadeq Ali-Hasan-Al-Saegh, Antonia Annegret Jauken, Prokopis-Andreas Zotos, Thanos Athanasiou, Stefan Ruemke, Jan Karsten, Jawad Salman, Fabio Ius, Ezin Deniz, Bastian Schmack, Arjang Ruhparwar, Alina Zubarevich, Alexander Weymann

**Affiliations:** 1 Department of Cardiothoracic, Transplantation and Vascular Surgery, Hannover Medical School, Carl-Neuberg-Straße 1, 30625 Hannover, Germany; 2 Department of Cardiothoracic Surgery, Oxford Heart Centre, John Radcliffe Hospital, Oxford University NHS Foundation Trust, Oxford OX3 9DU, UK; 3 Department of Cardiac Surgery, Thessaly University, 38221 Larissa, Greece; 4 Department of Anaesthesiology and Intensive Care Medicine, Hannover Medical School, 30625 Hannover, Germany

**Keywords:** minimally invasive aortic valve replacement, transcatheter aortic valve implantation, aortic stenosis, elderly

## Abstract

**Background/Objectives:** Transcatheter aortic valve implantation (TAVI) has become the leading treatment option for patients suffering from aortic valve stenosis aged over 70, except in cases of specific contraindications like bicuspid valves, inappropriate access routes, or endocarditis. Minimally invasive aortic valve replacement (MIAVR) has emerged as a potential way to combine the durability of surgery with reduced procedural trauma. This study aims to assess the safety and feasibility of MIAVR in elderly patients. **Methods:** A total of 990 patients were included in this retrospective cohort study. Among them, 261 (26%) were aged 70 years or older (elderly cohort), and 729 (74%) were younger than 70 years (younger cohort). All patients were followed for at least 30 days postoperatively, with survival data collected through May 2025. Multivariable logistic regression, linear regression, and Kaplan–Meier survival analyses were performed. **Results:** Elderly patients were more likely to be female (51% vs. 40%, *p* = 0.001) and carried a heavier burden of vascular and renal comorbidity: renal impairment 33% vs. 17% and extracardiac arteriopathy 45% vs. 30% (both *p* < 0.001). Major bleeding occurred more frequently in the elderly cohort (7.7% vs. 4.1%; *p* = 0.02), as did new permanent pacemaker implantation (10% vs. 5.8%; *p* = 0.021) and sepsis (3.4% vs. 1.1%; *p* = 0.012). Rates of stroke, perioperative myocardial infarction, ECMO/right-heart failure, re-thoracotomy, and postoperative dialysis were low and comparable across age groups (all *p* > 0.20). Overall, 30-day mortality was 2.4% (24/990), with crude mortality approximately threefold higher among patients aged ≥70 years (4.6% vs. 1.6%). **Conclusions:** Our findings indicate that MIAVR is a feasible and safe surgical option across age groups; Elevated morbidity in elderly patients is primarily due to bleeding, pacemaker implantation, and sepsis, while rates of stroke, renal failure, and myocardial infarction are low.

## 1. Introduction

Severe aortic valve disease, particularly aortic stenosis, is increasingly common in the aging population and remains a major cause of morbidity and mortality [1,2]. With rising life expectancy, more patients requiring valve intervention are elderly, often with significant comorbidities and reduced physiological reserve [1,2,3]. In this context, the choice of treatment strategy is critical. Current guideline recommendations designate TAVI as the first-line treatment for patients aged ≥70 years, regardless of their operative risk [4]. Surgical aortic valve replacement continues to represent a durable and reliable therapy [5,6]. Minimally invasive aortic valve replacement (MIAVR) has emerged as a potential way to combine the durability of surgery with reduced procedural trauma [1,2].

MIAVR, most often performed via partial upper ministernotomy or right anterior thoracotomy, avoids full sternotomy while providing adequate access for valve replacement [3,4,5,6,7]. Reported benefits include decreased bleeding, lower transfusion requirements, reduced postoperative pain, shorter intensive care and hospital stays, and improved cosmetic results [1,2,3,4,5]. These advantages may be particularly important for elderly patients, who are more susceptible to prolonged recovery and postoperative complications after conventional sternotomy.

Despite these potential benefits, the application of MIAVR in older age groups remains debated. Critics argue that limited surgical exposure may prolong cardiopulmonary bypass and cross-clamp times, potentially increasing perioperative risk [5,6]. Elderly patients, with their greater vulnerability to ischemia and frailty-related complications, might be especially affected by such factors [5,6]. The key clinical question is whether the benefits of reduced surgical trauma outweigh the potential disadvantages of longer operative times and technical challenges in this high-risk population.

Available evidence suggests that MIAVR is generally safe, with outcomes comparable to conventional surgery, even in elderly cohorts. However, most prior studies have been limited by small sample sizes, heterogeneous populations, and inconsistent age definitions [7,8,9]. Few investigations have specifically compared outcomes between younger and elderly patients within large, uniform cohorts.

The present study seeks to address this gap by evaluating the safety and feasibility of MIAVR across different age groups in a large, contemporary patient population. By stratifying outcomes according to age, this work provides insights into whether MIAVR remains a suitable surgical option for elderly patients facing aortic valve replacement. We acknowledge that TAVI has become the leading treatment option for patients aged over 70, except in cases of specific contraindications like bicuspid valves, inappropriate access routes, or endocarditis. Moreover, while TAVI is often prioritized, it is essential to further explore the role of MIAVR in older patients, especially in instances where TAVI cannot be performed due to these contraindications.

## 2. Materials and Methods

### 2.1. Study Population

From January 2012 to April 2025, a total of 1200 consecutive patients underwent minimally invasive aortic valve replacement (MIAVR). After excluding cases with incomplete datasets, 990 patients were included in the final analysis. Among these, 261 patients (26%) were aged 70 years or older (elderly cohort), while 729 patients (74%) were younger than 70 years (younger cohort). A total of 210 patients were excluded from our analysis due to incomplete data or missing information regarding long-term follow-up. All procedures were carried out through a partial upper ministernotomy approach. Clinical and perioperative data were obtained from the institutional SAP database. The underlying etiologies of aortic valve disease comprise degenerative, ischemic, rheumatic, and infective origins.

### 2.2. Ethical Statement

In accordance with German regulations, formal approval from an ethics committee was not required, as the present study was conducted retrospectively and did not involve any interventional procedures.

### 2.3. Surgical Technique

All operations were performed using a standardized minimally invasive approach through a 5–7 cm upper sternotomy, either in J-shaped or reverse T-shaped configuration. After pericardial exposure, cardiopulmonary bypass was established via direct aortic and right atrial cannulation or femoral cannulation, depending on individual patient anatomy. After induction of cardiac arrest, the ascending aorta was opened, and the diseased aortic valve was excised. Valve replacement was carried out with either a bioprosthetic or mechanical prosthesis, secured using standard suturing techniques. The aorta was subsequently closed, the heart de-aired and reperfused, and anticoagulation reversed with protamine. Chest drains were placed as required, and the sternotomy was closed in anatomical layers.

### 2.4. Follow-Up and Patient Data Collection

All patients were followed for at least 30 days after surgery, with survival data collected through May 2025. Postoperative outcomes were systematically recorded, including the occurrence of extracorporeal membrane oxygenation (ECMO) use or right heart failure, major bleeding events, new pacemaker implantation, myocardial infarction, dialysis, re-thoracotomy, sepsis, and stroke. In addition, the duration of intensive care unit (ICU) stay and total length of hospitalization were documented. Early mortality was defined as death occurring during the index hospitalization or within 30 days of surgery. Late mortality was defined as any death occurring beyond the initial 30-day postoperative period. The 30-day and long-term follow-up for patients was conducted through electronic health records.

### 2.5. Statistical Analysis

All statistical analyses were performed using SPSS software (version 28, IBM Corp., Armonk, NY, USA). Continuous variables are presented as mean ± standard deviation (SD), while categorical variables are reported as absolute numbers and percentages. Group comparisons were carried out using the Mann–Whitney U test for continuous variables, and the Chi-square or Fisher’s exact test for categorical variables, as appropriate. Initially, univariable analyses were performed to evaluate the individual effect of each predictor variable on the outcome (Supplemental Table S1). Multivariable logistic regression was employed to identify independent predictors of postoperative complications and mortality, while linear regression was applied for continuous outcome measures. Survival analysis for all-cause mortality was performed using Kaplan–Meier methods, and survival curves were plotted for the longest follow-up period. A *p*-value of less than 0.05 was considered statistically significant, and all statistical tests were conducted as two-sided.

## 3. Results

### 3.1. Demographic Data

Between January 2012 and February 2025, 990 consecutive patients with aortic valve stenosis underwent MIAVR and met all inclusion criteria. Of these, 261 (26%) were aged ≥70 years (elderly cohort) and 729 (74%) were aged < 70 years (younger cohort). Age, by definition, differed markedly between groups (83.6 ± 3.2 years vs. 67.4 ± 10.7 years). Elderly patients were more likely to be female (51% vs. 40%, *p* = 0.001) and carried a heavier burden of vascular and renal comorbidity: renal impairment 33% vs. 17% and extracardiac arteriopathy 45% vs. 30% (both *p* < 0.001). They also presented more often in symptomatic heart-failure class (NYHA III–IV, 26% vs. 18%, *p* < 0.001). In contrast, the prevalence of COPD, diabetes, and pre-operative dialysis did not differ significantly (all *p* > 0.15). These patterns indicate that chronological age clustered with end-organ vascular disease rather than with pulmonary or metabolic comorbidity (Table 1).

### 3.2. Postoperative Morbidity

Early non-fatal complications demonstrated selective age-related patterns. Major bleeding was significantly more common in the elderly cohort (7.7% vs. 4.1%; *p* = 0.02), as was the need for a new permanent pacemaker (10% vs. 5.8%; *p* = 0.021) and sepsis (3.4% vs. 1.1%; *p* = 0.012). Rates of stroke, perioperative myocardial infarction, ECMO/right-heart failure, re-thoracotomy, and postoperative dialysis were low and did not differ by age (all *p* > 0.20). Length-of-stay metrics showed only modest differences: mean ICU stay was 2.7 ± 3.8 days in the elderly cohort versus 2.2 ± 3.4 days in younger patients (*p* = 0.040), while overall hospital stay was identical (median 14 days for both groups, *p* = 0.6). These data suggest that haemorrhagic and infective events account for most of the excess morbidity in the elderly rather than neurologic or renal complications (Table 2).

### 3.3. Intrahospital Mortality

Intrahospital death occurred in 32 of 923 evaluable patients (3.5%). On multivariable analysis, renal impairment was the strongest determinant (adjusted OR = 2.44, 95% CI 1.13–5.28; *p* = 0.022). Age ≥ 70 years showed a borderline association (OR = 2.08, 95% CI 0.99–4.35; *p* = 0.053); COPD trended similarly but did not reach significance (OR = 2.02, *p* = 0.097). Sex and extracardiac arteriopathy were neutral (both *p* > 0.20) (Table 3). Thus, while chronological age influences very early outcomes, pre-existing renal dysfunction more strongly governs in-hospital mortality after adjustment.

### 3.4. Early (30-Day) Mortality

Overall, 30-day mortality was 2.4% (24/990). Crude rates were threefold higher in the ≥70-year cohort (4.6% vs. 1.6%). In a multivariable logistic model adjusting for sex, COPD, renal impairment, and extracardiac arteriopathy, age ≥ 70 years remained an independent predictor of early death (adjusted OR = 2.38, 95% CI 1.02–5.52; *p* = 0.044). None of the other covariates reached statistical significance (all *p* ≥ 0.14), underscoring the dominant influence of chronological age on perioperative survival (Table 4).

### 3.5. Long-Term Survival

Median follow-up was 6.7 years (inter-quartile range 4.2–9.1 years), providing 5685 patient-years of observation. A total of 43 deaths accrued (4.3% of the cohort): 22 among elderly patients and 21 among younger patients.

**Figure 1:**
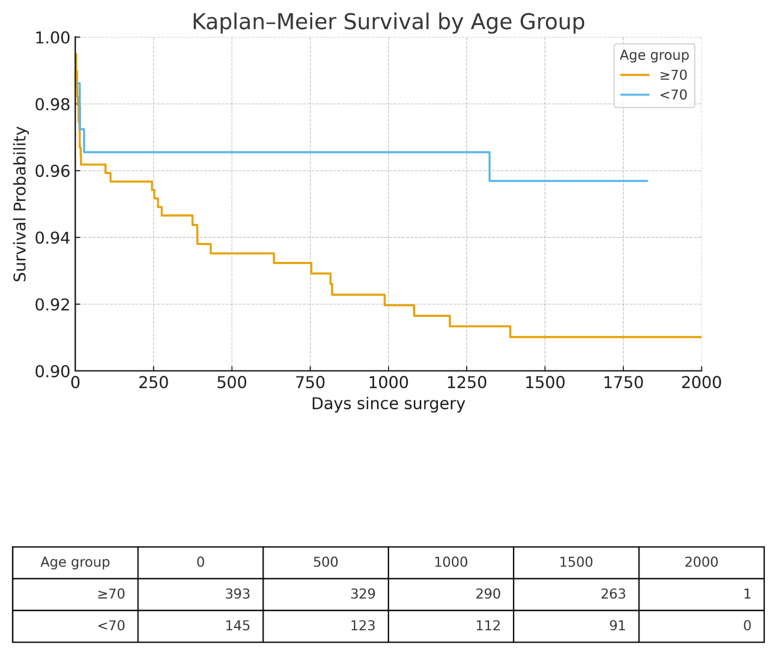
Kaplan–Meier analysis.

Kaplan–Meier analysis (Figure 1) showed an early and persistent separation of the survival curves. Three-year survival was 92% in patients ≥ 70 years vs. 97% in those <70 years, a difference that remained significant by the log-rank test (χ^2^ = 12.1, *p* < 0.001).

In the Cox proportional-hazards model (Table 5)—which incorporated the same covariates used for early mortality—age ≥ 70 years conferred a 2.5-fold increase in hazard of death (HR = 2.48, 95% CI 1.33–4.64; *p* = 0.004). COPD emerged as a second independent predictor (HR = 2.19, 95% CI 1.09–4.42; *p* = 0.028), whereas renal impairment exhibited a borderline effect (HR = 1.88, *p* = 0.066). Sex and extracardiac arteriopathy were not associated with late mortality.

## 4. Discussion

### 4.1. Summary of Key Findings

In this large contemporary cohort of patients undergoing MIAVR, we observed that elderly patients experienced significantly worse outcomes than their younger counterparts. Patients aged ≥70 years demonstrated a threefold increase in 30-day mortality (4.6% vs. 1.6%), and advanced age remained an independent predictor of early death even after multivariable adjustment (OR 2.38, 95% CI 1.02–5.52). Long-term survival also differed substantially, with the elderly cohort showing a two-and-a-half-fold increased hazard of death compared to younger patients (HR 2.48, 95% CI 1.33–4.64). Postoperative morbidity followed a similar age-related pattern, with major bleeding, permanent pacemaker implantation, and sepsis occurring more frequently in older patients, while neurologic and renal complications remained relatively uncommon across age groups. Importantly, renal impairment was the strongest determinant of in-hospital mortality, highlighting the interplay between age and comorbidity. Taken together, these findings confirm that while MIAVR can be performed safely across age groups, advanced age exerts a dominant influence on perioperative and long-term outcomes.

According to current guidelines, surgical aortic valve replacement is the preferred option for individuals under 70 years of age, provided they have a low surgical risk. Conversely, for patients aged 70 and older with a tricuspid aortic valve, TAVI is recommended as the primary treatment approach, assuming the anatomical conditions are suitable and transfemoral access is feasible. This strategy aims to reduce early complications and promote faster recovery [4].

### 4.2. Postoperative Bleeding

In our cohort, major postoperative bleeding was significantly more frequent in elderly cases compared with younger patients (7.7% vs. 4.1%, *p* = 0.025). This age-related susceptibility likely reflects vascular fragility, diminished coagulation reserve, polypharmacy (notably antithrombotic therapy), and impaired endothelial function. Although minimally invasive cardiac surgery is often promoted for reduced morbidity, our findings underscore that bleeding remains a major concern in elderly patients.

These observations are consistent with prior literature. A large multicenter study by Francica et al. demonstrated that elderly patients (>75 years) undergoing minimally invasive mitral valve surgery (MIMVS) had significantly higher rates of bleeding complications, including re-exploration, transfusion, and prolonged ICU stay, even after propensity matching [10]. Similarly, Cocchieri et al. reported a higher incidence of reintervention for bleeding among elderly patients undergoing endoscopic MIMVS when valve replacement was performed versus repair (10.9% vs. 0%, *p* = 0.005), implicating procedural complexity as an additional risk factor [11].

In a propensity-matched analysis, Hisatomi et al. found that major complications, including bleeding, were strongly associated with delayed ambulation and hospital discharge in patients aged ≥70 years, despite no difference in overall mortality [12]. Likewise, Elhassan et al. observed that although mortality was not elevated in elderly patients undergoing MICS with retrograde perfusion, bleeding-related complications such as transfusion and reoperation occurred more frequently, particularly in those with comorbidities or elevated EuroSCORE II [13].

Tabata et al. conducted a study involving 146 redo MIAVR in an elderly population. Among these, the majority, comprising 93 patients, had a history of coronary artery bypass grafting. The operative mortality rate was found to be 4.1%, while the incidence of reoperations due to bleeding was 0.7%. Additionally, a significant proportion of patients (83.6%) required blood transfusions [14]. Elbardisi et al. reported a 4% incidence of reoperation for postoperative bleeding among octogenarians who underwent MIAVR [15].

### 4.3. Permanent Pacemaker Implantation

PPI occurred significantly more often in elderly cases than in younger patients in our study (6.6% vs. 2.6%, *p* = 0.014). This finding is consistent with existing evidence and highlights the interplay between age-related conduction system vulnerability and intraoperative manipulation during minimally invasive valve surgery.

Data from the Mini-Mitral International Registry support this association. Among 7513 patients undergoing MIMVS with or without concomitant tricuspid valve repair, PPM implantation rates were higher in those undergoing concomitant tricuspid valve repair, particularly older patients (9% vs. 5.8%, *p* = 0.02) [16]. Independent predictors included age, mitral valve replacement, and atrial fibrillation surgery [16].

Earlier evidence from Stevenson et al. showed a 30-day complication rate of 5.4% following PPM implantation in patients aged ≥70 years, with higher all-cause 30-day mortality reflecting frailty rather than procedural complexity [17]. Complementing this, Bujak-Rogala et al. found that although pacemaker implantation improved quality of life (QoL) and reduced frailty symptoms in elderly patients, advanced age remained a negative predictor of postoperative QoL across all domains [18].

Newer device strategies may mitigate some of these risks. Marschall et al. reported that leadless pacemakers in elderly patients were associated with fewer complications, such as infection and lead dislodgement, compared with traditional transvenous systems [19].

### 4.4. Postoperative Sepsis

Our study found a notably higher rate of sepsis among elderly cohorts compared to younger cohorts following MIAVR, suggesting age-related vulnerability to infectious complications even in less invasive procedures. This observation is consistent with previous research highlighting both the benefits and residual risks of MICS in older populations.

A landmark study by Grossi et al. comparing minimally invasive port-access surgery (MIPA) to full sternotomy in elderly valve patients found that the MIPA group had significantly lower rates of sepsis and wound complications (1.8% vs. 7.7%, *p* = 0.027), demonstrating that the reduced surgical trauma associated with MICS indeed mitigates—but does not eliminate—the risk of postoperative infections in this group [20].

Hisatomi et al. conducted a propensity-matched comparison of elderly patients undergoing valve surgery via mini-thoracotomy vs. full sternotomy and found comparable in-hospital mortality, but significantly improved recovery metrics in the mini-thoracotomy group. Importantly, major complications—including sepsis—were strong predictors of delayed ambulation, highlighting the clinical burden of infectious events in elderly surgical recovery [12].

Elhassan et al. showed that retrograde femoral perfusion—a common approach in MICS—did not increase infection or neurological risks in elderly patients when compared to younger cohorts, reinforcing the notion that the surgical technique itself is safe but must be balanced against host-related risk factors such as immunosenescence and comorbidity burden [13].

Lastly, Petersen et al. also found that elderly patients undergoing minimally invasive valve surgery had outcomes equivalent to younger patients in terms of mortality and infection, provided proper risk stratification was performed. However, the elderly still showed slightly higher rates of complications overall, again reflecting a residual age-associated susceptibility [21].

Collectively, these findings suggest that while MICS offers reduced infectious risks compared to traditional approaches, elderly patients remain uniquely predisposed to sepsis and related complications due to inherent physiological limitations. Vigilant perioperative care and infection control protocols remain essential in this subgroup.

### 4.5. Stroke and Renal Failure

Stroke and acute kidney injury are feared complications following cardiac surgery, particularly in elderly patients due to decreased physiological reserves and increased vascular comorbidities. While minimally invasive cardiac surgery reduces surgical trauma, it may not fully mitigate these risks, particularly in older populations [22].

In our cohort, although stroke rates were low, they were still more prevalent in the elderly group compared to younger patients. A large retrospective analysis by Franz et al. on patients aged ≥75 undergoing minimally invasive mitral valve surgery found no statistically significant difference in stroke incidence between older and younger groups (3% vs. 0.6%, *p* = 0.16), though a higher trend in the elderly remained evident [23].

Importantly, the use of preoperative CT screening to guide femoral cannulation has been shown to reduce perioperative stroke. A meta-analysis by Leonard et al. revealed a significantly lower stroke incidence in patients undergoing preoperative CT-based surgical planning compared to those without (1.5% vs. 2.2%, *p* = 0.03), suggesting that imaging-guided perfusion strategies may be particularly beneficial in elderly patients undergoing MICS [24].

Acute renal failure is another critical concern in elderly patients post-cardiac surgery. Our findings indicated a modestly higher rate of renal failure in elderly cohorts. Consistent with this, Hage et al. conducted a meta-analysis comparing MIMVS and conventional sternotomy in elderly patients and found significantly lower odds of AKI in the MIMVS group (OR 0.27, 95% CI 0.10–0.78), reinforcing MICS as a renal-protective strategy [7].

Furthermore, the extensive review by Chertow et al. found that acute renal failure requiring dialysis after cardiac surgery was independently associated with increased early mortality (adjusted OR 7.9, 95% CI 6–10), underscoring the importance of even modest postoperative renal impairment in elderly surgical patients [22].

Collectively, these findings align with our results, suggesting that although MICS reduces overall risk, elderly patients remain susceptible to stroke and renal injury, which can substantially influence morbidity and mortality. Thus, strategies such as preoperative CT screening, renal protective measures, and meticulous intraoperative management remain essential to improving outcomes in this high-risk cohort. We advocate for future research to thoroughly assess the role of MIAVR in elderly patients who have contraindications to TAVI, including conditions such as bicuspid valves, unsuitable access routes, and endocarditis.

### 4.6. Key Clinical Message

Importantly, renal impairment emerged as a stronger predictor of in-hospital mortality than chronological age, underscoring the need to integrate both comorbid burden and physiologic reserve into surgical decision-making. Taken together, our results indicate that MIAVR should not be withheld solely on the basis of age, but rather offered selectively to elderly patients following thorough risk stratification. Future research should focus on refining perioperative protocols—particularly for bleeding control, infection prevention, and pacing management—to optimize outcomes in this growing patient population.

### 4.7. Limitations

This study is limited by its observational, single-center design, which may restrict generalizability and leave room for residual confounding despite multivariable adjustment. The lack of a sternotomy control group prevents direct comparison of MIAVR with conventional approaches in the elderly. In addition, dichotomizing age at 70 years, while clinically pragmatic, may overlook more nuanced gradations of risk. A further limitation of this study is the absence of detailed information on the implanted valve prostheses. Parameters such as valve type (mechanical vs. bioprosthetic), valve size, and whether a sutured or sutureless technique was used were not recorded in our institutional database and therefore could not be included in the analysis. The evaluation of postoperative outcomes following MIAVR was performed without a comparative arm involving conventional aortic valve replacement via full sternotomy. The lack of a sternotomy control group and TAVI cohort limits the ability to directly compare MIAVR with other surgical approaches in the elderly. Therefore, we recommend that future studies include a direct comparison between MIAVR and conventional aortic valve replacement, particularly for patients aged ≥70 years, to provide more informative and clinically relevant insights into the optimal management of this patient population. Finally, although median follow-up exceeded six years, very long-term outcomes beyond a decade were not assessable, particularly in the elderly cohort.

## 5. Conclusions

Our findings indicate that MIAVR is a feasible and generally safe option for patients across various age groups. However, elderly patients (aged ≥70 years) exhibit significantly worse outcomes, with a threefold increase in 30-day mortality and a 2.5-fold higher risk of late death, even after adjusting for comorbidities. The heightened morbidity in this group is mainly attributed to bleeding, pacemaker implantation, and sepsis, while rates of stroke, renal failure, and myocardial infarction remain low and comparable to those of younger patients.

## Figures and Tables

**Table 1:** Baseline characteristics.

Variable	<70 Years	≥70 Years	*p*-Value
Age (years)	67.4 ± 10.7	83.6 ± 3.2	<0.001
Female sex	291/729 (39.9%)	134/261 (51.3%)	0.001
BMI (kg/m^2^)	27.5 ± 6.3	26.2 ± 4.6	0.004
COPD	89/729 (12.2%)	41/261 (15.7%)	0.151
Renal impairment	125/729 (17.1%)	85/261 (32.6%)	<0.001
On dialysis pre-op	14/729 (1.9%)	9/261 (3.4%)	0.160
Extracardiac arteriopathy	217/729 (29.8%)	118/261 (45.2%)	<0.001
Diabetes (any)	174/729 (23.9%)	61/261 (23.4%)	0.871
NYHA III–IV	118/666 (17.7%)	68/257 (26.5%)	<0.001

**Table 2:** Postoperative complications by age group.

Complication	<70 Years	>70 Years	*p* Value
ECMO/right heart failure	4/729 (0.5%)	2/261 (0.8%)	0.698
Hospital stay (days)	14.9 ± 6.5	16.4 ± 11.6	0.605
ICU stay (days)	2.2 ± 3.4	2.7 ± 3.8	0.04
Major bleeding	30/729 (4.1%)	20/261 (7.7%)	0.025
New pacemaker	42/729 (5.8%)	26/261 (10.0%)	0.021
Myocardial infarction	1/729 (0.1%)	1/261 (0.4%)	0.448
Dialysis	13/729 (1.8%)	8/261 (3.1%)	0.217
Re-thoracotomy	22/729 (3.0%)	9/261 (3.4%)	0.732
Sepsis	8/729 (1.1%)	9/261 (3.4%)	0.012
Stroke	13/729 (1.8%)	3/261 (1.1%)	0.486

**Table 3:** Logistic regression for intrahospital mortality.

Covariate (Row Order)	Log HR	HR (95% CI)	*p*	Interpretation
Age ≥ 70 years	0.909	2.48 (1.33–4.64)	0.004	The elderly cohort has ~2.5-fold higher long-term mortality vs. those <70 years, independent of other factors.
Male sex	−0.257	0.77 (0.42–1.43)	0.411	Sex is not a significant predictor after adjustment.
COPD	0.785	2.19 (1.09–4.42)	0.028	COPD roughly doubles the risk of death.
Renal impairment	0.631	1.88 (0.96–3.69)	0.066	Trend toward higher risk, but not quite significant at 0.05.
Extracardiac arteriopathy	−0.081	0.92 (0.47–1.80)	0.813	No evident association with late mortality.

**Table 4:** Multivariable logistic regression on 30-day mortality.

Variable	OR	95% CI L	95% CI U	*p* > |z|
Age ≥ 70 years	2.377842	1.024619	5.518277	0.043739
Male sex	1.173534	0.506475	2.71915	0.70897
COPD	1.733351	0.648504	4.632979	0.272827
Renal impairment	1.9436	0.793203	4.762438	0.146139
Extracardiac arteriopathy	0.995601	0.409587	2.420054	0.992239

**Table 5:** Cox proportional-hazards model for overall survival.

Covariate	Hazard Ratio	95% CI	*p*
Age ≥ 70 years	2.48	1.33–4.64	0.004
COPD	2.19	1.09–4.42	0.028
Renal impairment	1.88	0.96–3.69	0.066
Male sex	0.77	0.42–1.43	0.411
Extracardiac arteriopathy	0.92	0.47–1.80	0.813

## Data Availability

Data is provided within the manuscript or Supplementary Information Files.

## Supplementary Materials

The following supporting information can be downloaded at https://www.mdpi.com/article/10.3390/jcm15010354/s1. Table S1. Univariate Analysis.
