# Supplementary material for: Minimally Invasive Aortic Valve Replacement in Elderly Patients: Insights from a Large Cohort
Source: J Clin Med. 2026 Jan 2;15(1):354. doi: 10.3390/jcm15010354 (PMC12786521; doi:10.3390/jcm15010354)
Supplement: Supplementary file 1 [file jcm-15-00354-s001.zip › jcm-3993686-supplementary.pdf]

Supplemental Table S1. Univariate Analysis

**Univariate Analysis on 30 day mortality**			
**Variable**	**OR**	**95% CI**	**p-value**
Age ≥70 years	1.91	0.71–5.17	0.201
Male sex	1.17	0.52–2.67	0.702
COPD	2.08	0.81–5.35	0.127
**Renal impairment**	**2.50**	**1.09–5.71**	**0.030**
Extracardiac arteriopathy	1.50	0.67–3.39	0.328
Diabetes (any)	1.24	0.52–2.92	0.630
**NYHA III–IV**	**3.50**	**1.54–7.95**	**0.003**
**Univariate Analysis on intrahospital mortality**			
**Age ≥70 years**	**2.75**	**1.05–7.23**	**0.039**
Male sex	1.62	0.77–3.40	0.202
**COPD**	**2.49**	**1.13–5.51**	**0.024**
**Renal impairment**	**3.15**	**1.54–6.42**	**0.002**
Extracardiac arteriopathy	1.57	0.78–3.20	0.209
Diabetes (any)	1.50	0.72–3.11	0.279
**NYHA III–IV**	**4.84**	**2.37–9.89**	**<0.001**
**Univariate survival analysis (all-cause mortality)**			
**Variable**	**HR†**	**95% CI**	**p-value (log-rank)**
Age ≥70 years	2.12	0.89–5.04	0.095
Male sex	0.85	0.46–1.59	0.469
**COPD**	**2.74**	**1.37–5.48**	**0.019**
**Renal impairment**	**2.94**	**1.57–5.51**	**0.001**
Extracardiac arteriopathy	1.28	0.68–2.39	0.412
Diabetes (any)	1.76	0.92–3.37	0.100
**NYHA III–IV**	**4.74**	**2.53–8.88**	**<0.001**
